# Affective Theory of Mind as a residual ability to preserve mentalizing in amnestic Mild Cognitive Impairment: A 12-months longitudinal study

**DOI:** 10.3389/fneur.2022.1060699

**Published:** 2022-11-18

**Authors:** Federica Rossetto, Sara Isernia, Monia Cabinio, Alice Pirastru, Valeria Blasi, Francesca Baglio

**Affiliations:** IRCCS Fondazione Don Carlo Gnocchi ONLUS, Milan, Italy

**Keywords:** Social Cognition, affective and cognitive Theory of Mind, Alzheimer's disease continuum, Mild Cognitive Impairment, longitudinal design, rehabilitation

## Abstract

**Introduction:**

Theory of Mind (ToM) decline has been outlined in people with amnestic Mild Cognitive Impairment (aMCI), but evidence from longitudinal studies is lacking. This longitudinal study aims to investigate changes in cognitive and affective ToM performance in an aMCI sample (*n* = 28; 14 females, mean age = 76.54 ± 4.35).

**Method:**

Participants underwent two steps of neurocognitive evaluation, at the baseline (T1) and 12-month follow-up (T2), to obtain their global cognitive level and both affective (Reading the Mind in the Eyes test, ET) and cognitive (Strange Stories, SS) ToM profile. Then, participants were categorized into two groups based on ToM changes: people who worsened (ET_Δ < 0_; SS_Δ < 0_) and people who did not (ET_Δ≥0_; SS_Δ≥0_) at follow-up. Differences between groups in cognitive functions and ToM profiles at baseline have been investigated.

**Results:**

Our results showed that 46% of subjects worsened in affective (ET) and 28% in cognitive (SS) ToM at follow-up. People who worsened in ET reported a statistically significantly higher performance in ET at baseline (*p* = 0.002) but not at follow-up than people who did not worsen. In contrast, subjects who worsened in SS showed a lower Immediate Free Recall (IFR, *p* = 0.026) and Delayed Free Recall (DFR, *p* = 0.028) score of the Free and Cued Selective Reminding test at baseline and at follow-up, a lower ET (*p* = 0.020) baseline score, a lower SS and MMSE level at follow-up than people who not worsened. About 71% of MCI subjects showed the same trend of evolution of the Mini-Mental State Examination and SS. Variables that significantly differed between groups have been inserted in a stepwise logistic regression to pilot explore predictors of affective and cognitive ToM evolution. Logistic regression showed ET at baseline (*p* = 0.015) as the only significant predictor of affective ToM evolution (R^2^ = 0.450), while both ET (*p* = 0.044) and memory performance (*p* = 0.045) at baseline significantly predicted cognitive ToM evolution (R^2^ = 0.746).

**Discussion:**

In conclusion, our results support the role of affective ToM as a residual mentalizing ability in preserving the mentalizing level in people with aMCI.

## Introduction

“We are an essentially social species” (1). Much of our behavior in everyday life is motivated by social goals from the early stage of infancy. For that reason, Social Cognition constitutes a crucial neurocognitive ability in the entire life span that allows us to process and interpret social information such as other people's intentions, feelings, and thoughts (2). Theory of Mind (ToM), or mentalizing, represents one of the most investigated key components of Social Cognition. It refers to the ability to understand, explain and predict own and others' behaviors on the basis of complex mental states such as beliefs and desires (3). ToM constitutes a multidimensional construct requiring the integration of cognitive (cold) as well as affective (hot) ToM processing (4–7). Specifically, cognitive ToM represents the ability to understand own and others' intentions, beliefs, and thoughts, while affective ToM concerns reasoning about own and others' affective states, emotions, or feelings.

Different trends of evolution in affective and cognitive ToM have been highlighted with advancing age. Experimental research in this field highlighted a complex, multifaceted picture of gain and loss, at least partially independent of age-related cognitive decline (8). Results from cross-sectional studies showed that older people perform poorly than younger adults in both cognitive and affective ToM regardless of task types and administration modality (9). Reduced performance in older individuals has been detected with the Reading the Mind in the Eyes test [ET, (10)], one of the most used tasks for the evaluation of the affective ToM (11–15). Cognitive ToM shows similar age effects, with older adults performing worse than younger in the advanced cognitive ToM tasks (9). Selective failures in cognitive or affective ToM have also been detected in aging conditions such as neurodegenerative pathologies (16).

A loss of mentalizing skills constitutes a hallmark feature of the Alzheimer's disease (AD) continuum, especially in the advanced ToM competencies [for a review, see (17)]. Although poorly characterized, a decline in mentalizing has also been outlined in people with amnestic Mild Cognitive Impairment (aMCI) (18), a transitional phase from successful aging to dementia (19). Impaired social functioning in the preclinical stage of AD holds high functional and clinical relevance since, with the progression of the disease, it may result in early social behavior changes, loss of independence in daily life, and poor quality of life. A recent meta-analysis (17) highlighted that both cognitive and affective ToM was reduced in a sample of 197 aMCI patients. However, there is only a little evidence of ToM changes in neurodegenerative conditions. Our previous pilot study on an aMCI cohort suggested a critical role of affective ToM in combating the conversion in dementia: individuals showing a worsening in general cognitive functioning were likely to improve in the ET test at 1-year follow-up (20). However, mechanisms underlying different patterns of affective and cognitive ToM changes in neurodegenerative conditions should be better explored and understood.

This longitudinal study aims to: (1) investigate 1-year-changes in cognitive and affective ToM performance; (2) disentangle the association between ToM and global cognitive changes; (3) explore possible predictors of ToM evolution in an aMCI sample.

## Materials and methods

### Participants

Twenty-eight outpatients diagnosed with aMCI were consecutively recruited at the Memory Clinic of IRCCS Don Carlo Gnocchi Foundation by the neurologist during the periodical examination in the clinic, based on the following inclusion criteria: (1) diagnosis of MCI due to AD according to the recommendations of the National Institute on Aging (21) and the DSM-5 diagnostic criteria (American Psychiatric Association, 2013); (2) age >65 years; (3) school attendance ≥ 5 years; (4) memory complaint relative to age-matched and education-matched healthy people, confirmed by an informant and documented over time by at least two consecutive steps of neuropsychological examination (21, 22) precedent to the beginning of the present study; (5) mostly preserved activities of daily living; (6) absence of psychiatric illnesses and severe auditory/visual impairment.

The study conforms to the ethical principles of the Helsinki Declaration revised (2008), with the approval from the Don Gnocchi local ethics committee. Informed written consent was obtained by all participants before taking part in the study.

### Procedure

After recruitment, the aMCI subjects underwent two steps of evaluation, at the baseline (T1) and 12 months after the baseline (T2), including a neurocognitive battery to obtain their cognitive and ToM profile. At each evaluation step (T1 and T2), participants were involved in an individual session of about 1 h.

#### Cognitive profile

The global cognitive level was investigated through the Mini-Mental State Examination [MMSE, (23)]. The total score was adjusted for age and education according to the Italian normative data (24); episodic memory was assessed with the Free and Cued Selective Reminding test [FCSRT, (25)]. The following total scores were computed and adjusted for sex, age and education according to Frasson et al. (25): the Immediate Free Recall (IFR) and the Delayed Free Recall (DFR); the lexical performance has been evaluated with the Phonemic Fluency test [FAS, (26)]; the abstract reasoning was assessed with the Raven's Progressive Matrices (27).

#### ToM profile

Two ToM tasks were administered to evaluate the affective and cognitive components of mentalizing. The Reading the Mind in the Eyes test [ET, (10)], validated in its Italian version (28, 29), was used to evaluate the attribution of affective mental states. Participants were invited to infer the mental states depicted in 36 eye gaze photographs in which only the eye region was visible, choosing among 4 words describing complex feelings or thoughts under each picture. Each item is scored 1 (correct) or 0 (wrong) for a total score range from 0 to 36. The same stimuli were administered with different instructions, detecting the correct gender of the person depicted in the photographs, as a control task (Gender test–GT, range 0–36). The Strange Stories task [SS, (30)], a valid test of ToM (31), was administered to assess the cognitive ToM level. A selection of 4 mentalistic stories was used for the purposes of the present study. In detail, in line with previous works (14, 20, 32–34), 4 mentalistic stories requiring the understanding of cognitive mental states in different social situations (persuasion, white lie, double bluff, and misunderstanding) have been selected. The 4 stories with the shortest text have been considered to minimize the influence of cognitive load on the ToM performance of people with aMCI. For the same purpose, the text remained visible while participants answer the SS questions.

The stories were consequently read to subjects, who were invited to answer questions about story comprehension, states of mind detection, and reasoning. Each story received a score of 0 for wrong answers, 1 for partially correct answers, and 2 for correct answers. The global scores of both mentalistic and physical stories ranged from 0 to 8.

### Statistical analysis

#### Participants' description

Frequencies, mean, and standard deviation of variables at T1 and T2 were extracted. Paired comparison (paired *t*-test or Wilcoxon test when appropriate) was run to report eventual statistically significant changes at follow-up.

#### Detection of the profile of participants worsening and who did not in ToM at follow-up

For ToM measures, a delta score (Δ) was computed to detect changes in the performance between T1 and T2. Then, participants were categorized into two groups based on the Δ score of ET and SS: people who worsened (Δ < 0; ET_Δ−_, SS_Δ−_) and people who did not (Δ ≥ 0; ET_Δ+/=_, SS_Δ+/=_) at follow-up. Descriptive statistics and unpaired comparisons (independent *t*-test or Mann-Whitney test or Chi-squared test when appropriate) were run to investigate differences in demographics, cognitive, and ToM profiles at baseline and at follow-up between the ET_Δ+/=_ / SS_Δ+/=_ and the SS_Δ+/=_ / SS_Δ−_ group.

#### Exploration of the association between ToM and global cognitive changes

For the MMSE score, a delta score (Δ) was computed, and participants were categorized into people who worsened in MMSE (Δ < 0; MMSE_Δ−_) and who did not (Δ ≥ 0; MMSE_Δ+/=_). Then, contingency tables were computed to test the contingency between changes at MMSE and ET, between MMSE and SS, and between ET and SS.

#### Exploration of predictors on ToM evolution at follow-up

Binary logistic regression classification models were computed including demographical characteristics (sex and age), global cognitive level at baseline, ToM test performance at baseline, and other variables at baseline statistically significantly different in the previous unpaired comparisons as possible predictors on ToM changes (ET and SS) at 12-month follow-up. Wald forward option was used as a stepwise selection method.

JASP (version 0.16.1) was used for all the statistical analyses.

## Results

### Participants

Twenty-eight subjects performed the evaluation at baseline and follow-up and were included in the analysis. Table 1 shows the demographical characteristics, cognitive, and ToM profiles of the participants at the baseline (T1) and follow-up (T2, 12 months from T1). According to Italian normative data, the ET mean score of the whole aMCI sample was borderline, with an equivalent score of 1 (range 15.73–19.23). Conversely, the GT mean score was within the normal range (33.43 ± 2.54 at T1 and 33.71 ± 2.24 at T2), with an equivalent score of 2 (range 32.76–34.25) (35).

**Table 1:** Participants' characteristics at T1 and T2, and comparison results.

		**Test range (cut-off)**	**T1**	**T2**	**Paired comparison *t (p)***
Demographics	N	-	28	28	
	Sex (Ma:F)	-	14:14	-	
	Age (y) (M, sd)	-	76.54, 4.35	-	
	Education years (M, sd)	-	10.64, 3.52	-	
Cognitive profile	MMSE (M, sd)	0–30 (23.80)	27.30, 1.89	26.53, 3.71	161.50^∧^(0.259)
	IFR (M, sd)	0–36 (19.59)	20.51, 6.01	20.74, 8.30	0.28^§^ (0.779)
	DFR (M, sd)	0–12 (6.31)	6.36, 4.02	6.43, 4.58	114.00^∧^ (0.474)
	FAS (M, sd)	–(17.35)	31.83, 11.38	31.14, 12.58	0.51^§^ (0.615)
	Raven (M, sd)	0–36 (18.00)	27.41, 5.11	28.12, 4.65	124.50^∧^ (0.473)
ToM profile	ET (M, sd)	0–36	18.64, 6.12	18.75, 4.94	0.123^§^(0.903)
	SS (M, sd)	0–8	4.25, 1.96	4.79, 1.99	1.16^§^(0.256)

DFR, Delayed Free Recall; ET, Reading the Mind in the Eyes Test; FAS, Phonemic Fluencies; GT, Gender Test; IFR, Immediate Free Recall; M, mean; MMSE, Mini-Mental State Examination; sd, standard deviation; SS, Strange Stories; Y, years.

§ = paired sample t-test was performed;

∧ = Wilcoxon test was performed.

### ToM changes at follow-up

46% of subjects worsened in ET (ET_Δ−_ group), and 28% in SS (SS_Δ−_ group). Tables 2, 3 show the demographic, cognitive, and ToM profile at baseline of people who worsened at ToM (ET_Δ−_ and SS_Δ−_ group) vs. people who did not (ET_Δ+/=_, SS_Δ+/=_). The ET_Δ−_ group reported a statistically significantly higher performance than the ET_Δ+/=_ group in ET at baseline (*p* = 0.002, Cohen's d = 0.67). The SS_Δ−_ group showed a lower IFR (*p* = 0.026, Cohen's d = 0.52), DFR (*p* = 0.028, rank biserial correlation = 0.54), and ET (*p* = 0.020, Cohen's d = 1.03) score than SS_Δ+/=_ group at baseline, and a lower MMSE (*p* = 0.013, rank biserial correlation = 0.61), IFR (*p* = 0.005, Cohen's d = 1.29), DFR (*p* = 0.021, rank biserial correlation = 0.57), and SS (*p* = 0.002, Cohen's d = 0.76) than SS_Δ+/=_ group at follow-up.

**Table 2:** Comparison of demographics, cognitive, and ToM profile of ET_Δ−_ and ET_Δ+/=_ group.

		**ET_Δ−_**	**ET_Δ+/=_**	**Unpaired comparison (*p*)**
	**N**	**13**	**15**	
	**ET Δ (M, sd)**	**−3.92, 2.98**	**3.60, 2.26**	**7.57^§^ (<0.001)**
Demographics	Sex (Ma:F)	6:7	8:7	0.14° (0.705)
	Age (y) (M, sd)	75.15, 4.06	77.73, 4.37	1.61^§^ (0.119)
	Education (y) (M, sd)	11.46, 2.11	9.93, 4.35	1.15^§^ (0.259)
Cognitive profile	MMSE at T1 (M, sd)	27.29, 1.83	27.31, 2.00	98.00^∧^ (1.00)
	MMSE at T2 (M, sd)	26.82, 4.38	26.29, 3.14	126.50^∧^ (0.188)
	IFR at T1 (M, sd)	19.43, 5.73	21.44, 6.28	0.88^§^ (0.386)
	IFR at T2 (M, sd)	19.13, 7.82	22.14, 8.72	0.96^§^ (0.347)
	DFR at T1 (M, sd)	5.85, 3.62	6.80, 4.41	83.50^∧^ (0.533)
	DFR at T2 (M, sd)	6.18, 4.22	6.64, 5.00	94.00^∧^ (0.890)
	FAS at T1 (M, sd)	32.00, 11.15	31.69, 11.97	0.07^§^ (0.944)
	FAS at T2 (M, sd)	31.81, 14.60	30.55, 11.02	0.26^§^ (0.797)
	Raven at T1 (M, sd)	27.36, 5.85	27.46, 4.60	99.00^∧^ (0.963)
	Raven at T2 (M, sd)	28.58, 5.32	27.73, 4.13	114.50^∧^ (0.447)
ToM profile	ET at T1 (M, sd)	22.31, 4.33	15.47, 5.74	3.51^§^ (0.002)
	ET at T2 (M, sd)	18.38, 4.33	19.07, 5.55	0.36^§^ (0.723)
	SS at T1 (M, sd)	3.92, 1.98	4.53, 1.96	0.82^§^ (0.421)
	SS at T2 (M, sd)	4.85, 2.15	4.73, 1.91	0.15^§^ (0.884)

DFR, Delayed Free Recall; ET, Reading the Mind in the Eyes Test; F, females; IFR, Immediate Free Recall; M, mean; Ma, males; MMSE, Mini-Mental State Examination; sd, standard deviation; SS, Strange Stories;

§ = unpaired sample t-test was performed;

∧ = Mann-Whitney test was performed;

° = Chi-squared test was performed.

**Table 3:** Comparison of demographics, cognitive, and ToM profile of SS_Δ−_ and SS_Δ+/=_ group.

		**SS_Δ−_**	**SS_Δ+/=_**	**Unpaired comparison *(p)***
	**N**	**8**	**20**	
	**SS Δ (M, sd)**	**−2.25**	**1.67**	**5.352^§^ (<0.001)**
Demographics	Sex (Ma:F)	3:5	11:9	0.70° (0.403)
	Age (y) (M, sd)	77.25, 4.89	76.25, 4.22	0.54^§^ (0.592)
	Education (y) (M, sd)	9.37, 4.34	11.15, 3.12	1.22^§^ (0.235)
Cognitive profile	MMSE at T1 (M, sd)	26.77, 2.32	27.51, 1.70	60.00^∧^ (0.319)
	MMSE at T2 (M, sd)	23.44, 5.21	27.77, 1.98	31.00^∧^ (0.013)
	IFR at T1 (M, sd)	16.60, 7.24	22.07, 4.79	2.35^§^ (0.026)
	IFR at T2 (M, sd)	14.05, 7.53	23.42, 7.11	3.10^§^ (0.005)
	DFR at T1 (M, sd)	3.58, 3.36	7.47, 3.77	36.50^∧^ (0.028)
	DFR at T2 (M, sd)	3.35, 3.89	7.66, 4.31	34.00^∧^ (0.021)
	FAS at T1 (M, sd)	29.16, 12.50	32.90, 11.07	0.78^§^ (0.443)
	FAS at T2 (M, sd)	27.86, 13.21	32.45, 12.42	0.87^§^ (0.393)
	Raven at T1 (M, sd)	25.06, 5.66	28.35, 4.70	52.50^∧^ (0.170)
	Raven at T2 (M, sd)	25.69, 4.87	29.09, 4.30	49.00^∧^ (0.121)
ToM profile	ET at T1 (M, sd)	14.50, 4.44	20.30, 5.99	2.47^§^ (0.020)
	ET at T2 (M, sd)	16.12, 4.26	19.80, 4.89	1.86^§^ (0.075)
	SS at T1 (M, sd)	5.12, 1.88	3.90, 1.92	1.53^§^ (0.137)
	SS at T2 (M, sd)	2.87, 1.96	5.55, 1.43	4.02^§^ (0.002)

DFR, Delayed Free Recall; ET, Reading the Mind in the Eyes Test; F, females; FAS, Phonemic Fluencies; IFR, Immediate Free Recall; M, mean; Ma, males; MMSE, Mini-Mental State Examination; sd, standard deviation; SS, Strange Stories;

§ = unpaired sample t-test was performed;

∧ = Mann-Whitney test was performed;

° = Chi-squared test was performed.

39.29% of subjects reported the same trend of changes in MMSE and ET at 12-month follow-up (7 subjects maintained both MMSE and ET performance at follow-up, while 4 subjects showed a worsening in both tests) (Table 4). Conversely, 71.43% of subjects showed the same trend at the follow-up in SS and MMSE (14 subjects maintained their performance level at both tests, while 6 subjects worsened at both ones) (Table 5). Finally, 39% of subjects had the same response at ET and SS at follow-up (9 subjects maintained both SS and ET performance at follow-up, while 2 subjects showed a worsening in both tests) (Table 6).

**Table 4:** Contingency table of people who worsened/not worsened both at Mini-Mental State Examination (MMSE) and ET.

	**ET_Δ+/=_ *N* (%)**	**ET_Δ−_** ***N* (%)**	**Total *N* (%)**
MMSE_Δ+/=_	7 (25.00)	9 (32.14)	16 (57.14)
MMSE_Δ−_	8 (28.57)	4 (14.29)	12 (42.86)
Total	15 (53.57)	13 (46.43)	28 (100.00)

MMSE, Mini-Mental State Examination.

**Table 5:** Contingency table of people who worsened/not worsened both at Mini-Mental State Examination (MMSE) and SS.

	**SS_Δ+/=_ *N* (%)**	**SS_Δ−_** ***N* (%)**	**Total *N* (%)**
MMSE_Δ+/=_	14 (50.00)	2 (7.14)	16 (57.14)
MMSE_Δ−_	6 (21.43)	6 (21.43)	12 (42.86)
Total	20 (71.43)	8 (28.57)	28 (100.00)

MMSE, Mini-Mental State Examination.

**Table 6:** Contingency table of people who worsened/not worsened both at Reading the Mind in the Eyes Test (ET) and SS.

	**SS_Δ+/=_ *N* (%)**	**SS_Δ−_** ***N* (%)**	**Total *N* (%)**
ET_Δ+/=_	9 (32.14)	6 (21.43)	15 (53.57)
ET_Δ−_	11 (39.29)	2 (7.14)	13 (46.43)
Total	20 (71.43)	8 (28.57)	28 (100.00)

ET, Reading the Mind in the Eyes Test.

### Predictors of ToM changes at follow-up

The binary logistic regression model including sex, age, MMSE, and ET score at baseline as possible predictors of ET changes (stepwise method), highlighted ET at baseline as the only predictor of changes in affective ToM at follow-up (Table 7; Δχ^2^ = 11.50, *p* = <0.001). High performance in ET at T1 was associated with a high probability of worsening in ET at follow-up.

**Table 7:** Binary logistic regression model to test best predictors of ET change at follow-up.

**Model**		**Estimate**	**SE**	**OR**	**z**	**p**	**95% CI (lower, upper)**	**Nagelkerke R^2^**	**AUC**
1	Intercept	0.143	0.379	1.154	0.378	0.706	−0.60, 0.89	-	0.833
2	ET at T1	−0.323	0.133	0.724	−2.43	0.015	−0.58,−0.062	0.450	
	Intercept	6.321	2.633	555.89	2.40	0.016	1.16, 11.48		

95% CI, 95% confidence interval; ET, Reading the Mind in the Eyes Test; OR, Odd Ration; SE, Standard Error.

Four logistic regression models were computed (stepwise method) including sex, age, ET, SS, MMSE, and DFR at baseline as possible predictors of cognitive ToM changes. The best model (model 4) highlighted DFR at T1 and ET at T1 as significant predictors of changes in SS at follow-up (Δχ^2^ Model 1–2 = 5.98, *p* = 0.015. Δχ^2^ Model 2–3 = 8.26, *p* = 0.004. Δχ^2^ Model 3–4= 6.34, *p* = 0.012; Table 8). High performance in DFR and ET at baseline was associated with the probability of not worsening in SS at follow-up.

**Table 8:** Binary logistic regression model to test best predictors of SS change at follow-up.

**Model**		**Estimate**	**SE**	**OR**	**z**	***p***	**95% CI (lower, upper)**	**Nagelkerke R^2^**	**AUC**
1	Intercept	0.916	0.418	2.500	2.190	0.028	0.10, 1.74	-	0.969
2	DFR at T1	0.287	0.132	1.332	2.169	0.030	0.03, 0.55	0.275	
	Intercept	−0.494	0.720	0.610	−0.686	0.492	−1.90, 0.92		
3	DFR at T1	0.479	0.235	1.614	2.034	0.042	0.02, 0.94	0.571	
	ET at T1	0.388	0.200	1.474	1.937	0.053	−0.00, 0.78		
	Intercept	−7.965	4.128	0.000	−1.930	0.054	−16.05, 0.12		
4	DFR at T1	0.766	0.383	2.152	2.000	0.045	0.02, 1.52	0.746	
	ET at T1	0.488	0.242	1.629	2.018	0.044	0.01, 0.96		
	SS at T1	−1.210	0.668	0.298	−1.812	0.070	−2.52, 0.10		
	Intercept	−5.037	4.022	0.006	−1.252	0.210	−12.92, 2.85		

95% CI, 95% confidence interval; DFR, Delayed Free Recall; ET, Reading the Mind in the Eyes Test; OR, Odd Ration; SE, Standard Error; SS, Strange Stories.

## Discussion

This study aimed to investigate longitudinal changes in mentalizing abilities in people with aMCI, focusing on both cognitive and affective components, disentangling their link with cognitive functions, and exploring potential predictors of ToM evolution. Interesting results arose from the comparison between people who worsened at ToM (ET_Δ−_ and SS_Δ−_ group) with people who did not (ET_Δ+/=_, SS_Δ+/=_). Approximately half of the aMCI participants worsened in ET (46%), according to previous literature reporting an age-related decline of affective ToM in people with aMCI (17, 20, 36), and 28% of subjects got worse in SS.

Interestingly, people who worsened in affective ToM and people who worsened in cognitive ToM showed distinct profiles. In fact, while people getting worse in affective ToM were characterized by a high ET performance at T1, people who worsened in SS presented a low ET as well as memory level at baseline. These distinct patterns of evolution in aMCI were confirmed by the contingency analysis showing that in 60% of cases cognitive and affective ToM changes followed an opposite trend. Concerning the evolution of affective ToM, despite the initial difference in ET performance between people who worsened and people who did not, no difference was observed at follow-up, with a comparable level of affective ToM. Moreover, affective ToM seems to be predictive of ToM evolution in aMCI, in terms of residual mentalizing ability.

Concerning the evolution of cognitive ToM, high ET scores together with a high level of memory at the baseline resulted in an increased probability of not getting worse in the SS test. The protective role of memory on cognitive ToM is not surprising. Previous works supported the notion that ToM deficit in the aMCI population is secondary to cognitive impairment and memory performance (37). Instead, the role of affective ToM as a residual mentalizing ability holds the greatest interest. This finding is in accordance with our previous evidence suggesting a possible worsening-counteracting influence of the affective ToM component on the cognitive part. In fact, affective ToM had a protective role both on cognitive ToM and global cognitive level (20).

Differently from cognitive ToM, for affective ToM we did not observe the role of cognitive factors on its evolution but only of the affective ToM itself at baseline. This result confirmed that the affective component of ToM is independent of the other neurocognitive domains. This evidence was also supported by our contingency results: ET and MMSE showed a separate evolution trend. However, it has to be considered that the two ToM tasks used to assess cognitive and affective ToM were different for stimuli modalities, verbal (stories) vs. visual (photographs), and potentially required a distinct level of cognitive load. Especially based on the vision of ToM as an interactive process spanning different cognitive abilities (38, 39), story-based tasks, such as SS, may involve different non-ToM cognitive processes than visual tasks, such as ET. Nevertheless, it has been increasingly proven that the deficit in the neurological condition is only partly explained by task modality specificity (2).

This work is not exempt from limitations. Our participants' group is reduced in size, and further evidence is needed to confirm the findings. In these terms, this study remains exploratory and did not allow running more complex analyses requiring powered design. Also, more than one follow-up would make the data more informative regarding changes over time in ToM. Moreover, the absence of a control group is an additional shortcoming of the study: a control group would have allowed qualifying the pattern of ToM evolution of aMCI in relation to successful aging evolution pattern. Future contributions may include a control group, and explore a wider time span, such as 5-years follow-up and the rate of AD-converter. In addition, our results should be taken with caution in light of the lack of studies supporting the validity and reliability of the Strange Stories task in the Italian population. Also, for the purpose of this study, a selection of stories was used, with a score range of 0-8, which could prevent us from sensitively detecting performance individual differences.

In conclusion, our results support the role of affective ToM in preserving the mentalizing level in people with aMCI. Further studies are needed to enhance awareness about the role of ToM in neurodegenerative conditions since poor social functioning has been linked to unsuccessful interpersonal relationships and, consequently, poor quality of life, and wellbeing. Our results suggest that the clinical assessment of neurodegenerative conditions may focus not only on the more established aspects of cognition that may be negatively affected in the AD continuum but also on the affective and cognitive components of ToM. The comprehension of the interdependence between these two components will allow focus training and rehabilitation interventions also on socio-cognitive skills, such as affective ToM, to counteract the decline in mentalizing abilities.

## Data availability statement

The raw data supporting the conclusions of this article will be made available by the authors, without undue reservation.

## Ethics statement

The studies involving human participants were reviewed and approved by Don Gnocchi Local Ethics Committee. The patients/participants provided their written informed consent to participate in this study.

## Author contributions

FB, FR, and SI conceived the study. FR collected data and carried out the study. SI performed the statistical analysis. FR and SI wrote the draft of the manuscript. VB, MC, AP, and FB substantively revised and edited the draft of the manuscript. All authors read and approved the final version of the manuscript.

## Funding

This work was supported by 5x1000 funds - 2020, Italian Ministry of Health.

## Conflict of interest

The authors declare that the research was conducted in the absence of any commercial or financial relationships that could be construed as a potential conflict of interest.

## Publisher's note

All claims expressed in this article are solely those of the authors and do not necessarily represent those of their affiliated organizations, or those of the publisher, the editors and the reviewers. Any product that may be evaluated in this article, or claim that may be made by its manufacturer, is not guaranteed or endorsed by the publisher.
